# Local Lidocaine–Prilocaine for Immunisation in Infants

**DOI:** 10.3390/vaccines12121329

**Published:** 2024-11-27

**Authors:** Claudia-Felicia Pop, Petronela Coblișan, Valentina Sas, Cătălina Drugă, Paraschiva Cherecheș-Panța

**Affiliations:** 1 Department of Mother and Child, Nursing Discipline, Iuliu Hațieganu University of Medicine and Pharmacy, 400124 Cluj-Napoca, Romania; felicia.pop@umfcluj.ro; 2 Third Pediatric Discipline, Faculty of Medicine, Iuliu Hațieganu University of Medicine and Pharmacy, 400124 Cluj-Napoca, Romania; sas.valentina@umfcluj.ro (V.S.); pusachereches@umfcluj.ro (P.C.-P.); 3 General Medical Assistance Program, Faculty of Medicine, Iuliu Hațieganu University of Medicine and Pharmacy, 400124 Cluj-Napoca, Romania; dcatalina8@yahoo.com

**Keywords:** lidocaine and prilocaine, vaccination, infant

## Abstract

**Introduction**: Lidocaine–prilocaine cream effectively reduces vaccination pain, improving vaccination adherence and advocating for its routine use in healthcare settings. **Methods**: This review used PRISMA guidelines and the PICOT format to structure the analysis. The focus was on paediatric patients aged 0–12 months requiring intramuscular vaccinations, comparing the application of lidocaine–prilocaine cream to other interventions or no treatment. Data sources included MEDLINE, PUBMED, EMBASE, BMC, Research GATE, and Cochrane Library. Studies were selected based on predefined inclusion and exclusion criteria, with data extracted regarding participant demographics, pain assessment scales, and study conclusions. The Cochrane Risk of Bias Tool assessed study quality, while statistical analyses evaluated the effectiveness of the intervention. **Results**: Fifty studies were identified, with nine ultimately included in the analysis, involving 704 participants. The primary outcome was the effectiveness of lidocaine–prilocaine cream in reducing pain after vaccinations. Results showed a significant mean difference of −1.719 in pain scores for infants treated with lidocaine–prilocaine compared to those receiving other interventions. Behavioural indicators, such as crying duration and latency to the first cry, favoured the lidocaine–prilocaine group. **Conclusions**: Lidocaine–prilocaine cream effectively reduces vaccination pain, supporting its routine use in healthcare settings. Engaging parents can enhance vaccination adherence. Training healthcare professionals on lidocaine–prilocaine cream application and pain scales is vital for improving patient satisfaction. Further research is needed to evaluate the long-term effects of lidocaine–prilocaine cream on vaccination experiences and infant behavioural health.

## 1. Introduction

During early childhood, children often experience pain and stress during medical procedures, such as vaccinations. Infants are particularly vulnerable, with limited understanding and expression of their feelings, making pain assessment challenging. Research indicates that painful stimuli can impair psychological and neurological development, leading to both short- and long-term negative effects [1]. Additionally, healthcare professionals face significant parental anxiety during these procedures, and part of their role is to inform parents about effective pharmacological and non-pharmacological pain relief options. One pharmacological option for local pain reduction is lidocaine–prilocaine cream. Applying this cream before vaccination may significantly reduce infants’ vocal and behavioural reactions, resulting in a more positive experience for the child, parents, and healthcare providers.

### 1.1. Vaccination in Infants

In Romania, several mandatory vaccines are administered intramuscularly to infants 0–12 months (Table 1) [2,3,4]. Calmette Guerrin vaccine (BCG) is recommended intradermally during the first 24 h after birth.

Understanding the potential side effects associated with vaccination is crucial for both parents and healthcare professionals. The most common side effects after vaccination are fever and local side effects such as pain, redness or even the appearance of a lump [3,5,6]. Intramuscular injection is associated with considerable pain and distress in infancy [7,8]. A recent systematic review described, besides injection pain, a fear of needles on the part of both the child and the parents as a relevant issue that might interfere with acceptance of vaccination [9]. Both injection pain and fear of needles occur during vaccination with a prevalence between 5 and 13% in the paediatric population [9]. Factors that can interfere with vaccination in children are related to parent concerns but also with medical system management and particular situations, as noticed during COVID-19 pandemic when a delay of vaccinations in children was noticed [10].

Parental stress regarding vaccination often stems from insufficient education about pain management strategies. Many parents express a desire for information on reducing their child’s discomfort during vaccinations [11]. This stress can adversely affect the parent–child relationship and overall caregiving ability [12].

### 1.2. Pain in Infants

The International Association for the Study of Pain (IASP) defines pain as “an unpleasant sensory and emotional experience associated with actual or potential tissue destruction”. This definition highlights the distinction between nociception, the physiological process of detecting harmful stimuli, and the subjective experience of pain. Although infants have pain receptors like those in adults [13], their ability to process and interpret pain is limited. The development of pain pathways in infants begins at approximately 22 weeks of gestation and matures by around 2 months of age. During this critical developmental stage, exposure to painful stimuli can lead to long-term cellular changes, affecting neurological and emotional development [1,14]. Painful interventions, such as venipuncture, pricking the heel to obtain capillary blood, and vaccinations are among the most distressing for infants [15].

### 1.3. Pain Perception in Infants

Infants’ pain experiences vary significantly across different developmental stages. In the first two months, infants exhibit limited responses to pain, primarily manifesting as reflexive movements or behavioural signs like frowning and crying. By four months, as their neurological and sensory systems develop, infants begin to show more deliberate responses to painful stimuli and may start associating certain stimuli with discomfort [16]. At a younger age, infants depend on parents for comfort when they are in a stressful situation and in pain [17].

### 1.4. Factors Influencing Pain Response

Piira et al. categorise factors influencing infants’ pain responses into distal and proximal factors. Distal factors include gestational age, type of delivery, birth weight, baby’s temperament, and history of painful medical procedures [18,19,20]. For instance, low birth weight and vaginal delivery can act as stressors for children [1]. Proximal factors encompass immediate influences, such as the behaviour of parents and healthcare staff, as well as the environment in which the procedure takes place [18]. Parental anxiety can heighten the child’s perception of pain, while a calming environment and empathetic healthcare providers can alleviate distress [21]. The behaviour of the medical staff plays an essential role. A calm and empathetic nurse and doctor can help create a sense of security, which lessens the child’s anxiety. The technique of administering an intramuscular vaccine, with the prolongation of the procedure, can lead to exaggerated behavioural reactions [1,18].

### 1.5. Assessment of Pain in Infants

Parents often underestimate their infants’ pain, particularly if they lack experience with medical procedures.

Pain assessment scales, such as the FLACC scale (Face, Legs, Activity, Cry, Consolability), PIPP scale (Premature Infant Pain Profile), NIPS scale (Neonatal/Infant Pain Scale), NFCS (Neonatal Facial Coding System), and MBPS (Modified Behavioral Pain Scale), are valuable tools for evaluating pain in non-verbal infants [18,22,23,24,25].

### 1.6. Pain-Reducing Methods

Both pharmacological and non-pharmacological methods exist to alleviate pain in infants during vaccination. Effective non-pharmacological strategies include positioning the child with the ventral part placed on the mother’s chest (recommended for 0–6 months of age) or with their back on the mother’s chest (for children of 6–12 months of age), cuddling, distraction techniques, skin-to-skin contact [17], or facilitated tucking, which consists of placing the child in a lateral or ventral decubitus in a position similar to the position the fetus has in the last trimester of pregnancy [26,27]. Non-nutritive sucking, using a pacifier [28] or breastfeeding are outstanding non-pharmacological interventions for alleviating procedural pain in newborns. Breast milk is a unique elixir of comfort; the act of breastfeeding triggers the release of endorphins, nature’s own pain relievers, and fosters a crucial bond between mother and child, strengthening feelings of trust and security in stressful moments [17,29].

Pharmacological options range from sucrose or glucose solutions to topical anaesthetics like lidocaine–prilocaine cream, EMLA (Eutectic Mixture of Local Anaesthetics), or tetracaine gel [30,31,32].

Lidocaine–prilocaine cream, or EMLA, contains lidocaine 2.5% and prilocaine 2.5% in a 1:1 ratio, two amide-type active substances with a local anaesthetic effect. Its application can significantly reduce pain during medical procedures, although care must be taken to avoid potential side effects, such as local reactions or rare systemic effects [33].

The place where the procedure will be carried out must be determined with the nurse or doctor who will perform the procedure, checking that there are no traumatic injuries. Establishing the dose of the cream depends on the age and weight of the child and the complexity of the medical act.

This systematic review aims to consolidate current evidence on the use of lidocaine–prilocaine for pain management in infant immunisation procedures. It will address clinical challenges, implementation strategies for anaesthetic cream use, pain assessment scales, and future research implications. By enhancing the understanding and application of these methods, we can improve the immunisation experience for infants and their families.

## 2. Materials and Methods

This systematic review used the PRISMA (the Preferred Reporting Items for Systematic reviews) guidelines [34] and employed the PICOT framework to structure our evidence-based practice analysis. We focused on the following:

- Population (P): paediatric patients aged 0–12 months requiring intramuscular vaccination.
- Intervention (I): application of lidocaine–prilocaine cream to the thigh prior to vaccination.
- Comparison (C): effectiveness of the anaesthetic cream versus other interventions (placebo or no intervention).
- Outcomes (O): evaluation of whether lidocaine–prilocaine cream could effectively reduce vaccination pain, using pain scales (e.g., FLACC, NIPS), behavioural distress indicators, and physiological measures (e.g., heart rate).
- Types of studies (T): included randomised clinical trials, observational cohort studies, and relevant guidelines.

### 2.1. Data Sources and Search Strategies

We searched MEDLINE, PUBMED, EMBASE, BMC, ResearchGATE, and The Cochrane Library using terms like “EMLA cream” and “lidocaine–prilocaine”, combined with “infants”, “anaesthesia”, “immunisation”, and “vaccination”. Additional search terms included “pain management in infants” and “topical anaesthesia for immunisation”.

### 2.2. Study Selection

Eligible studies were selected based on titles and abstracts, followed by full-text reviews. The inclusion criteria mandated the following:

- Participants aged 0–12 months requiring intramuscular vaccination.
- Studies must involve lidocaine–prilocaine cream.
- Randomised controlled trials or comparative studies.
- Full-text articles published from 2000 to 2023.

Exclusion criteria eliminated studies involving patients over one year old, other medical procedures than vaccination, non-comparative designs, inadequate designs, non-English articles, and unpublished texts.

### 2.3. Data Extraction

Key data included age, participant numbers, vaccination indications, pain assessment metrics, and study conclusions. We employed the Cochrane Risk of Bias Tool for randomised trials to assess potential bias sources. Disagreements were resolved through consensus.

### 2.4. Data Analysis

The intervention’s effectiveness was evaluated by comparing pain scores between the anaesthetic cream group and others, using *t*-tests for statistical significance.

## 3. Results

**Figure 1:**
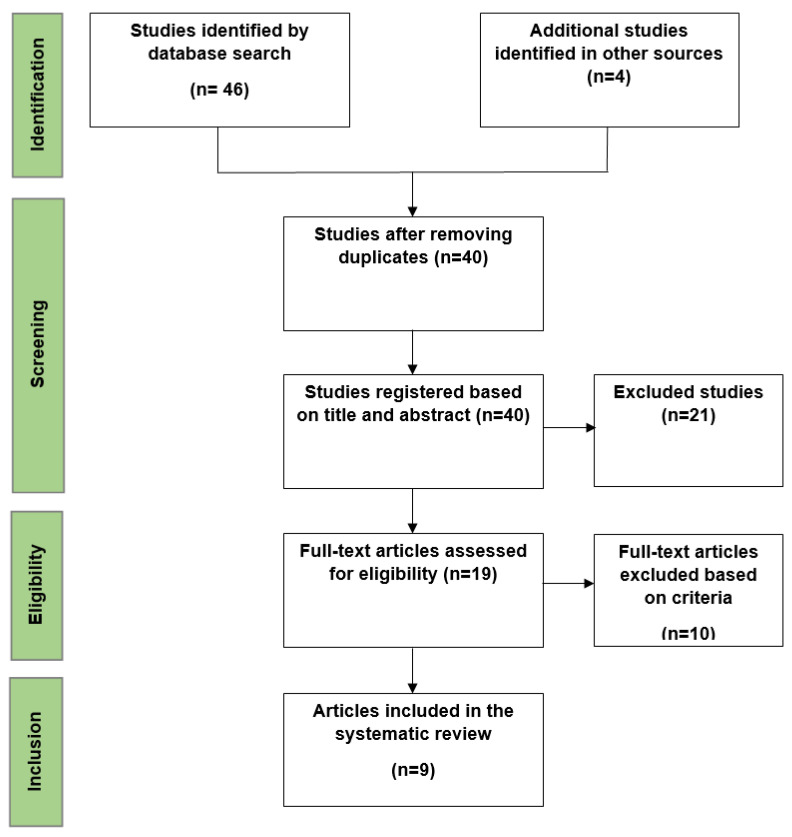
PRISMA flow chart of included and excluded studies.

Initially, 50 studies were identified, with 40 remaining after duplicates were removed. Title and abstract reviews excluded 21 studies, leading to the in-depth analysis of 19, ultimately resulting in 9 studies for final inclusion. Those stated have been graphically represented, below, based on the PRISMA flow chart (Figure 1).

- Selected Studies

The relevant characteristics of the analysed studies are summarised in Table 2, with the studies being noted chronologically, depending on the year of publication. Forty-one studies were excluded due to duplication, being studies in older children or adults [11,35,36], or being in language other than English (e.g., Korean, Arabic), leading to nine included studies. Most studies used 1 g of lidocaine–prilocaine cream, 60 min before vaccination, as the manufacturer recommends.

**Figure 2:**
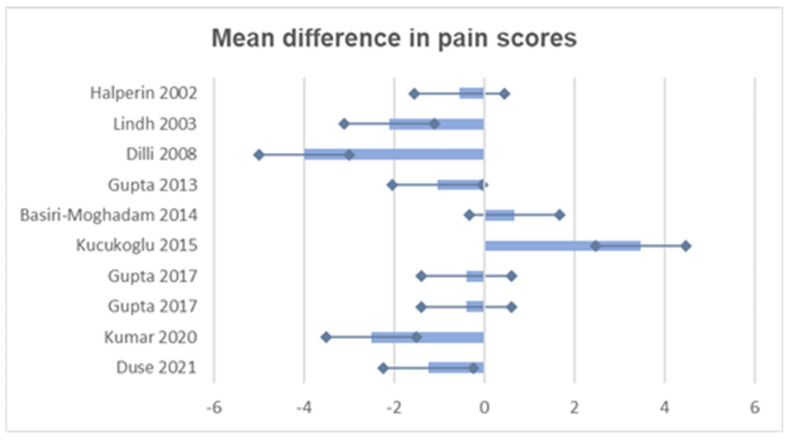
Mean difference in pain scores across included studies and 95% confidence interval [23,24,37,38,39,40,41,42,43].

All effect size estimates are associated with an assessment of statistical uncertainty, showing 95% confidence intervals and exact *p* values where available. The graph shown in Figure 2 illustrates the mean differences and confidence intervals of the pain scores from each study analysed, providing a picture of the precision and variability of the data.

- Study Population

The review comprised 704 participants aged 0–12 months, with 347 receiving the anaesthetic cream and 357 in the comparator group (patients who received placebo cream, other interventions or none—reference standard).

- Outcomes

Primary outcomes focused on the effectiveness of the lidocaine–prilocaine cream in reducing pain.

Secondary outcomes included pain assessment scores and behavioural indicators, such as total crying duration and first cry latency.

The results noted in Table 3 show a significant mean difference in pain scores, with a reduction of −1.719 (95% CI: −2.314 to −1.124) in infants who received lidocaine–prilocaine cream compared to those who received alternative interventions or no treatment. 

## 4. Discussion

This systematic review included nine studies that investigated the effects of using lidocaine–prilocaine cream in reducing pain associated with intramuscular vaccination in infants. The overall mean difference between lidocaine–prilocaine cream as compared with other pharmacological or non-pharmacological methods, or no intervention, indicates that lidocaine–prilocaine cream effectively alleviates vaccination-related pain.

The Modified Behavioral Pain Scale (MBPS) was the primary tool for assessing pain in non-verbal patients, although some studies also utilised the Visual Analog Scale (VAS) for parental assessment [38,43]. Key behavioral indicators measured included the latency of the first cry and total crying duration. Infants treated with lidocaine–prilocaine cream demonstrated a longer latency to the first cry and shorter crying duration compared to control groups (see Table 2). The total duration of crying was more prolonged in infants in the comparison groups. Lindh et al. (2003) noticed the fact that there were infants in the intervention group who did not cry at all, but their number did not exceed that of infants in the same group who did cry. Other scales might also be used, like FLACC or NIPS, for the most accurate evaluation of the impact of medical procedures on the mental and physical status of the infant. The use of scales can lead to improvement in medical practices.

While not all studies reported adverse effects of lidocaine–prilocaine cream, some noted mild reactions like erythema and local pruritus [40]. One study specifically assessed the impact of lidocaine–prilocaine cream on immune response to vaccines, finding no negative effects on antibody titers or seroconversion rates for various vaccine antigens [37]. Geometric mean antibody titers, seroconversion rates, or the proportion of participants achieving protective or positive titers after immunisation were not affected. For Pertussis, Diphtheria, Tetanus, Hepatitis B, and Haemophilus Influenzae type b antigens, antibody titers were similar between the lidocaine–prilocaine cream group and the placebo group, before and after 6 months from vaccination, respectively. A recent study aiming to investigate the systemic side effects of local anaesthetics (lidocaine, procaine, chloroprocaine, prilocaine, mepivacaine, bupivacaine, levobupivacaine, and ropivacaine) showed that 14 of 64 children that received topical lidocaine–prilocaine presented neurological symptoms (56.8%), cardiorespiratory symptoms (24.3%) or methemoglobinemia (18.9%) [33]. Neurological symptoms were mild, with most children presenting malaise, sleepiness, and hypotonia. Cardiorespiratory side effects included cyanosis, bradycardia, and hypoxia. This occurred in children to which parents administered excessive doses of lidocaine–prilocaine cream or patches.

The evidence supports lidocaine–prilocaine cream as a safe and effective option for reducing pain during vaccinations in infants, particularly in those aged 2–6 months [37,44]. However, in infants below 2 months old, there was no statistically significant differences in pain scores between lidocaine–prilocaine cream and the placebo group, indicating the need for further research in this younger demographic.

### 4.1. Applicability of Evidence

The appropriate management of pain experienced by infant patients during medical procedures is of major importance in paediatric clinical practice. The results of this systematic review, which demonstrated the effectiveness of lidocaine–prilocaine cream in alleviating pain during intramuscular vaccination, have the potential to positively influence how pain is managed in infants during routine vaccinations.

A recent study on the practice of nurses during infant immunisation revealed that topical anaesthetics were never used during immunisation in 90.4% of cases, or at most occasionally in 9.6% of situations [8]. Sweet solutions were not used in 90.4% of the cases, or they were administered occasionally in 6.4% of the cases. Breastfeeding or parent-/caregiver-led distraction were more common, but still 34.1%; respectively, 14.9% of infants did not benefit from these comforting non-pharmacological pain relief procedures [8].

The current evidence underlines the importance of developing a pain management protocol during medical procedures, including infant vaccination. There are several circumstances that need to be mentioned: the method of applying the cream, the waiting time before the vaccine is administered, the size of the needle, and differences in pain perception. It is essential that healthcare professionals follow standardised guidelines and protocols for the use of lidocaine–prilocaine cream to maximise the benefits of this intervention.

### 4.2. Concordances and Discordances

The results of this systematic review are consistent with the existing literature on the effects of lidocaine–prilocaine cream in reducing infant vaccination pain. Previous studies demonstrated the efficacy of this intervention in pain relief, confirming our findings that the use of lidocaine–prilocaine cream is superior as compared to a placebo or other non-pharmacological methods. The included studies indicate that the use of topical anaesthetics, such as lidocaine–prilocaine cream or cooling spray, are effective in reducing pain during vaccine administration in infants. The use of these methods led to more favorable results in terms of the pain score compared to those obtained with simple breastfeeding or other non-pharmacological techniques.

There are also some differences regarding the measured parameters. Some studies showed a significant reduction in crying duration in infants treated with lidocaine–prilocaine cream [40], while other studies found no significant differences in this parameter between the groups that used lidocaine–prilocaine cream and the comparator group [43]. Halperin et al. (2002) further reported that the use of lidocaine–prilocaine patches did not adversely affect the immune response to the vaccine, whereas the rest of the studies did not investigate this aspect.

### 4.3. Limitations

While most studies were of moderate to high quality, limitations included potential bias in randomisation processes and the insufficient blinding of staff, as expectations and perceptions of the intervention may influence subjective ratings of pain. Some studies did not adequately report the general health status of the patients. Associations with certain disorders could influence the perception of pain and, implicitly, the results obtained.

The variability in the application of the cream (e.g., the time between application of the cream and administration of the vaccine) and the demographic differences among infants might also have impacted the results.

## 5. Conclusions

This systematic review demonstrated that lidocaine–prilocaine cream effectively alleviates pain in infants undergoing intramuscular vaccinations. Its use should be considered standard practice in clinical settings. The implementation and use of pain scales by nurses in daily medical practice may improve the satisfaction of patients and their parents. Proper training for healthcare professionals on cream application and pain assessment is crucial to maximise its benefits. Moreover, the awareness and application of lidocaine–prilocaine cream highlight a significant area for future research, exploring its long-term impacts on vaccination experiences in all age groups.

## Figures and Tables

**Table 1:** Intramuscular vaccines in infants.

Type of Vaccine	Recommended Age
Hepatitis B vaccine (HB)	the first 24 h after birth
Diphtheria, Tetanus, Pertussis (acellular, component), Hepatitis B (rDNA), Poliomyelitis (inactivated) and Haemophilus Influenzae type B conjugate vaccine (adsorbed) (DTaP-IPV-HB-Hib)	2, 4, 11 months
Pneumococcal polysaccharide 13-valent conjugate vaccine	2, 4, 11 months
Measles, Mumps, and Rubella vaccine (MMR)	12 months

**Table 2:** Characteristics of the 9 included studies.

Author, Year, Country	Population	Intervention	Comparator	Vaccine	Scale Used	Behavioural Indicators	Conclusions
Halperin BA. et al., 2002, Canada [37]	153 infants aged between 0 and 6 months	EMLA cream patch (1 g 60–180 min before vaccination)	Patch with placebo cream	DTaP-IPV-Hib, Hepatitis B	MBPS	-	The highest statistically significant difference between pain scores was observed in infants aged 6 months. The group of patients aged 2–4 months was too small and no statistically significant differences between pain scores were identified. No difference was seen in 2-month-old infants.
Lindh V. et al., 2003, Sweden [38].	90 patients aged under 3 months	EMLA cream patch (1 g 60 min before vaccination) and glucose solution	Patch with placebo cream and water	DTaP	MBPS; VAS.	Presence of crying, latency to first cry, and total duration of crying.	Patients in the EMLA + glucose group had a significant reduction in pain scores, with less crying than in the placebo group, and crying started later and lasted less.
Dilli D. et al., 2008, Turkey [39].	14 patients aged between 6 and 12 months	Lidocaine–prilocaine cream (1 g 60 min before vaccination)	No intervention	Hepatitis B; MMR.	NIPS	Total duration of crying	The use of anaesthetic cream was much more effective than vaccination without any pre-applied intervention.
Gupta NK. et al., 2013, India [40].	60 patients aged under 3 months	EMLA cream patch (1 g 60 min before vaccination) and distilled water.	Placebo cream (Vaseline) and distilled water	DTaP	MFCS (modified NFCS scale)	Latency to first cry and total duration of crying.	The use of EMLA cream significantly reduced the pain score and total duration of crying in the group treated with it in combination with distilled water. The EMLA + breastfeeding group was not considered.
Basiri-Moghadam M. et al., 2014, Iran [41].	32 patients aged 4 months	EMLA cream (2 g 60 min before vaccination)	Rattle toy	Unspecified	Own scale, modified.	-	In the toy group, the pain was more severe than in the EMLA group. There was also a group that did not receive any intervention, and the pain score was significantly increased compared to the intervention groups.
Kucukoglu S. et al., 2015, Turkey [23].	75 patients aged 4 months	EMLA cream (0.5 g 30 min before vaccination)	Instrumental touch (placebo)	Hepatitis B	PIPP	-	The pain score in the EMLA group was lower than in the instrumental touch group.
Gupta NK. et al., 2017, India [24].	60 infants aged 3 months	EMLA cream (1 g 60 min before vaccination) and breastfeeding	Cooling spray with breastfeeding	DTaP	MFCS; NIPS.	Latency to first cry and total duration of crying.	The use of EMLA cream or cooling spray with breastfeeding reduced the pain score, but did not reduce the duration of crying.
Kumar M. et al., 2020, India [42].	150 patients aged between 6 weeks and 6 months	Lidocaine–prilocaine cream (no data regarding the dose, 60 min before vaccination)	Without intervention	Combined vaccine DTaP–HiB–Hepatitis B	MBPS		“The group that received the lidocaine–prilocaine cream had the lowest pain scores, followed by the groups that received other types of interventions, which were not included in our study”.
Duse BO. et al., 2021, Sweden [43].	70 patients aged 3 months	EMLA cream (1 g 60 min before vaccination)	Placebo cream	Pneumococcal	FLACC; VAS.	Latency to first cry and total duration of crying.	“The use of EMLA cream significantly reduced pain during vaccination, increasing the latency to the first cry; however, no significant reduction in the total duration of crying was observed”.

EMLA: Eutectic Mixture of Local Anaesthetics; g: gram; DTaP: Diphtheria–Tetanus–Pertussis acellular (DTPa); IPV: Inactivated Poliovirus Vaccine; Hib: Haemophilus Influenzae type B; NIPS: Neonatal Infant Pain Scale; PIPP: Premature Infant Pain Profile; FLACC: Face, Legs, Activity, Cry, Consolation; VAS: Visual Analog Scale; MBPS: Modified Behavioural Pain Scale; MFCS: Modified Face Cry Scale; NFCS: Neonatal Facial Coding System.

**Table 3:** Results of included studies.

Study	Mean Intervention (SD)	Mean Comparator (SD)	Mean Difference	Confidence Interval 95%	*p* Value
Halperin, 2002 [37]	2.77 (0.335)	3.33 (0.76)	−0.56	(−0.88, −0.24)	0.0029
Lindh, 2003 [38]	3.7 (1.8)	5.8 (1.8)	−2.1	(−3.5, −0.7)	0.004
Dilli, 2008 [39]	2.0 (1.02)	6.0 (0.51)	−4.0	(−5.2, −2.8)	0.001
Gupta, 2013 [40]	4.23 (2.66)	5.26 (1.83)	−1.03	(−2.08, 0.02)	0.05
Basiri-Moghadam, 2014 [41]	4.87 (1.31)	4.19 (1.94)	0.68	(−0.60, 1.96)	0.27
Kucukoglu, 2015 [23]	9.6 (5.12)	6.13 (5.97)	3.47	(1.54, 5.40)	0.001
Gupta, 2017 [24]	1.4 (2.4)	1.8 (2.5)	−0.4	(−1.59, 0.79)	0.05
	1.9 (3.1)	2.3 (3.0)	−0.4	(−1.98, 1.18)	0.05
Kumar, 2020 [42]	4.1 (1.1)	6.6 (0.9)	−2.5	(−3.1, −1.9)	0.0024
Duse, 2021 [43]	6.37 (2.22)	7.62 (1.48)	−1.25	(−2.11, −0.39)	0.006

(SD = standard deviation).
